# High infiltration of mast cells positive to tryptase predicts worse outcome following resection of colorectal liver metastases

**DOI:** 10.1186/s12885-015-1863-z

**Published:** 2015-11-04

**Authors:** Shinsuke Suzuki, Yasushi Ichikawa, Kazuya Nakagawa, Takafumi Kumamoto, Ryutaro Mori, Ryusei Matsuyama, Kazuhisa Takeda, Mitsuyoshi Ota, Kuniya Tanaka, Tomohiko Tamura, Itaru Endo

**Affiliations:** 1 Department of Gastroenterological Surgery, Yokohama City University Graduate School of Medicine, 3-9 Fukuura, Kanazawa-ku, Yokohama, 236-0004 Japan; 2 Department of Immunology, Yokohama City University Graduate School of Medicine, 3-9 Fukuura, Kanazawa-ku, Yokohama, 236-0004 Japan; 3 Department of Oncology, Yokohama City University Graduate School of Medicine, 3-9 Fukuura, Kanazawa-ku, Yokohama, 236-0004 Japan; 4 Department of Gastroenterological Surgery, Yokohama City University Medical Center, 4-57 Urafunecho, Minami-ku, Yokohama, 232-0024 Japan; 5 Department of Surgery, Teikyo University Chiba Medical Center, 3426-3 Anesaki, Ichihara, 299-0111 Japan

**Keywords:** Mast cells, Colorectal cancer, Metastasis, Survival

## Abstract

**Background:**

Accumulation of tumor-infiltrating mast cells (MCs) predicts poor survival in several cancers after resection. However, its effect on the prognosis of patients with colorectal liver metastases (CRLM) is not known.

**Methods:**

Our retrospective study included 135 patients who underwent potentially curative resection for CRLM between 2001 and 2010. Expression of tryptase, MAC387, CD83, and CD31, which are markers for MCs, macrophages, mature dendritic cells, and vascular endothelial cells, respectively, was determined via immunohistochemistry of resected tumor specimens. The relationship between immune cell infiltration and long-term outcome was investigated.

**Results:**

The median follow-up time was 48.4 months for all patients and 57.5 months for survivors. Overall survival (OS) rates at 1, 3, and 5 years were 91.0, 62.4, and 37.4 %, respectively. Five-year disease-free survival (DFS) and OS rates were 21.6 and 38.1 %, respectively, in patients with high MC infiltration, and 42.6 and 55.6 %, respectively, in patients with low MC infiltration (*p* < 0.01 for both DFS and OS). Infiltration of other types of immune cells did not correlate with survival. Multivariate analyses indicated that hypoalbuminemia and high peritumoral MC infiltration were significant predictors of unfavorable OS.

**Conclusion:**

High peritumoral MC infiltration predicts poor prognosis in patients who underwent hepatectomy for CRLM. The number of MCs in metastatic lesions is important for predicting the prognosis of CRLM patients and as an indication of therapy.

## Background

More than 1 million people are diagnosed with colorectal cancer (CRC) and approximately 0.5 million people die from this disease each year worldwide [1]. CRC is the second most common cancer in women and the third most common cancer in men [2]. Advanced CRC is frequently accompanied by synchronous or metachronous liver metastases [3]. Despite improvements in surgical techniques and the introduction of new chemotherapy agents, overall survival remains poor for most patients with colorectal liver metastases (CRLM) [4]. Five-year survival rates after hepatectomy are reported to range from 33 to 61 % [5–8].

Different types of infiltrating immune cells [mast cells, (MCs), macrophages (Mφs), dendritic cells (DCs), neutrophils, and lymphocytes] surround tumors in variable numbers and have different effects on tumor progression [9]. We reported the significance of tumor-infiltrating lymphocytes, such as regulatory T cells, as a predictor of worse outcome in CRLM patients [10]. Tumor-infiltrating MCs (TIMs) are considered a primary host immune response against cancer. However, their function varies among different cancers [11–17]. The function of TIMs near CRLM has never been reported.

Many tumors secrete stem cell factor (SCF), which attracts MCs to tumor sites [18]. Activation of the c-Kit pathway leads to MC activation and consequent expression of angiogenic cytokines [e.g., vascular endothelial growth factor (VEGF), platelet-derived growth factor (PDGF), and fibroblast growth factor 2 (FGF-2)] and tryptase-mediated MC degranulation [19, 20]. Tryptase is an agonist of protease-activated receptor-2 (PAR-2), expressed on vascular endothelial cells [21]. Activation of PAR-2 induces cell proliferation and release of interleukin 6 (IL-6) and granulocyte-macrophage colony stimulating factor, act as angiogenic molecules [22]. Therefore a strong positive correlation between MC density and microvascular density in many human and animal malignancies [23–34].

MCs are classically and conventionally identified via histochemical methods. Toluidine blue (Undritz stain) metachromatically stains MC granules red or blue-red owing to the presence of sulfated proteoglycans (e.g., heparin) [35]. MCs can be immunohistochemically stained with antibodies to c-Kit or proteins in their granules, such as tryptase or chymase. Primary anti-chymase and anti-tryptase antibodies produce diffuse cytoplasmic staining. Tryptase activates protease-activated receptor-2, and it’s activity stimulates proliferation of colon cancer cells [36]. Although TIMs may be a useful prognostic marker in CRC, their significance in CRLM is unclear. Therefore, the aim of the present study was to determine the prognostic significance of MC density in patients with CRLM.

## Methods

### Patients

Between January 2001 and December 2010, 258 patients with CRLM underwent initial liver resection at the Department of Gastroenterological Surgery at Yokohama City University Graduate School of Medicine. Patients who could not undergo curative resection (*n* = 61) or died during the immediate postoperative period (30 days) (*n* = 1) were excluded from our retrospective study. Patients with a pathologically complete response to neoadjuvant chemotherapy (NAC) (*n* = 4) were also excluded because evaluation of the initial cancer site in the liver was impossible. Of the remaining 192 patients, 135 with both clinicopathological data and resected specimens were analyzed. Primary lesion tissues were collected for 69 of the 135 patients. Preoperative staging, preoperative chemotherapy, hepatectomy procedures, adjuvant chemotherapy, and patient follow-up were previously described [37, 38]. 40 patients received oxaliplatin, and 17 patients received bevacizumab. The end of follow-up was defined as the time of the last follow-up (August 2014) or death. Informed consent for participation in the study was obtained from participants. Our study was approved by the Yokohama City University ethics committee.

### Immunohistochemistry

Tissue sections (4 μm thick) were deparaffinized in xylene and rehydrated through a series of graded alcohol. The endogenous peroxidase activity of the specimens was blocked by incubating the slides in absolute methanol containing 0.3 % hydrogen peroxide for 30 min at room temperature. Antigen retrieval was carried out via autoclave pretreatment (120 °C for 5 min) in citrate buffer (pH 6). After washing with phosphate-buffered saline, specimens were incubated with 10 % rabbit serum albumin for 10 min and primary antibody at 37 °C for 1 h.

**Fig. 1:**
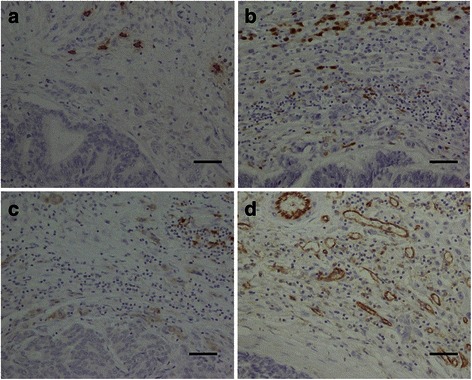
Representative immunohistochemical staining patterns of formalin-fixed, paraffin-embedded sections of colorectal liver metastases using monoclonal antibodies to (**a**) tryptase, (**b**) MAC387, (**c**) CD83, and (**d**) CD31. Original magnification, ×200. Scale bar, 50 μm

Mouse monoclonal antibodies were used to recognize tryptase (Abcam AA1, 1:100; MC marker), MAC387 (Abcam MAC387, 1:1000; Mφ marker), CD83 (Abcam 1H4b, 1:100; mature DC cell marker), and CD31 (Abcam JC 70A, 1:100; vascular endothelial cell marker). Immunohistochemical reactions were visualized using a HistoFine kit (Nichirei Pharmaceutical, Tokyo, Japan) and a DAB kit (Dako, Carpinteria, CA, USA). The sections were counterstained with hematoxylin and examined microscopically (Fig. 1a–d). As the negative control, Mouse IgG1 isotype control (monoclonal mouse IgG1 clone #11711; R&D Systems) was used at the same concentration, then all other steps were followed.

### Immunohistochemical evaluation

Evaluation was performed as previously reported [16, 33, 39, 40]. Cells were counted in each immune cell subset in the 3 most abundant peritumoral areas, and cell numbers were compared. “Peritumoral” was defined as the border between normal liver tissue and the tumor. Therefore we defined peritumoral area as a fields which include tumor three out of ten in view. “Micro vessel density” was defined as the number of blood vessels. Positively stained blood vessels with lumen as well as cell clusters without a lumen and single cells were considered as individual vessels [41]. Cell counting was performed by two investigators (SS and NK) without knowledge of the accompanying clinical information and under the supervision of a pathologist. In cases of discrepancy, a consensus was achieved using a multi-head microscope.

### Statistical analysis

The Kaplan-Meier method was used to estimate Overall survival (OS) and disease-free survival (DFS). To determine the optimal cutoff point, a receiver operating characteristic (ROC) curve was drawn for each variable, and the factor most highly correlated with OS was identified. The threshold was calculated by determining the Youden index [maximum (sensitivity + specificity - 1)] from the ROC curve [42]. Cox regression multivariate models were used to identify independent prognostic factors. The Mann-Whitney U and chi square tests were used to compare cell numbers between subgroups. Statistical significance was defined as *p* < 0.05. Analysis was performed using SPSS 20.0 software (SPSS Inc., Chicago, IL, USA).

## Results

### Patient characteristics

The clinicopathological features of patients enrolled in this study are summarized in Table 1. The patient group included 84 men and 51 women with an age range of 31–83 years (median 63 years). Most patients (67 %) had primary tumor nodal metastases, 58 % had synchronous liver metastases, and 24 % had extrahepatic disease at the time of hepatectomy. The median number of metastatic nodules was 4 (range 1–32), the median follow-up time was 48.4 months, and the minimum follow-up time was 17.8 months for survivors. DFS rates at 1, 3, and 5 years were 48.1, 32.6, and 20.7 %, respectively, for the entire group. OS rates at 1, 3, and 5 years were 91.0, 62.4, and 37.4 %, respectively.

**Table 1:** Clinicopathological charasteristics of 135 CRLM patients

Variable	All cases	Peritumoral MCs		*p* value
		Low	High	
Number of patients	135	62	73	
Age, years	63.4 ± 10.0	63.4 ± 10.1	63.5 ± 10.0	0.99
Sex [n (%)]				0.60
Male	84 (62.2)	37 (59.7)	47 (64.4)	
Female	51 (37.8)	25 (40.3)	26 (35.6)	
Location of primary tumor [n (%)]				0.90
Colon	89 (65.9)	41 (66.1)	48 (65.8)	
Rectum	46 (34.1)	21 (33.9)	25 (34.2)	
Primary lymph node metastases [n (%)]				0.58
Negative	44 (32.6)	22 (35.5)	22 (30.1)	
Positive	91 (67.4)	40 (64.5)	51 (69.9)	
Timing [n (%)]				0.06
Synchronous	78 (57.8)	30 (48.4)	48 (65.8)	
Metachronous	57 (42.2)	32 (51.6)	25 (34.2)	
Distribution [n (%)]				0.16
Uniloblar	56 (41.5)	30 (48.4)	26 (35.6)	
Biloblar	79 (58.5)	32 (51.6)	47 (64.4)	
Tumor number	5.7 ± 6.1	4.8 ± 5.6	6.4 ± 6.4	0.13
Maximum tumor size, mm	39.2 ± 24.6	38.3 ± 23.2	39.9 ± 25.8	0.70
Extrahepatic metastases [n (%)]				0.11
Present	33 (24.4)	11 (17.7)	22 (30.1)	
Absent	102 (75.6)	51 (82.3)	51 (69.9)	
Preoperative chemotherapy [n (%)]				0.30
Yes	70 (51.9)	29 (47.5)	41 (56.9)	
No	65 (48.1)	32 (52.5)	31 (43.1)	
Prehepatectomy CRP, mg/L	0.6 ± 1.3	0.7 ± 1.5	0.6 ± 1.2	0.62
Prehepatectomy Alb, g/dL	4.0 ± 0.4	4.0 ± 0.4	4.0 ± 0.5	0.53
Prehepatectomy CEA, ng/mL	201.1 ± 607.1	111.2 ± 341	277 ± 758.0	0.10
Peritumoral MCs	42.2 ± 36.8	11.2 ± 5.2	68.4 ± 31.1	<0.01
Normal liver areal MCs	13.6 ± 9.9	11.4 ± 8.6	15.5 ± 10.6	0.02
Peritumoral Mφs	113.2 ± 105.1	93.4 ± 99.1	130.0 ± 107.7	0.04
Peritumoral mature DCs	7.6 ± 8.7	8.2 ± 6.9	7.1 ± 10.0	0.43
Micro vessel density	39.6 ± 24.9	32.0 ± 16.6	46.0 ± 28.9	<0.01

*MCs* mast cells, *CRP* C-reactive protein, *Alb* albumin, *CEA* carcinoembryonic antigen, *M*φ*s* macrophages, *DCs* dendritic cells

### Tumor-infiltrating immune cells and clinicopathological features

**Fig. 2:**
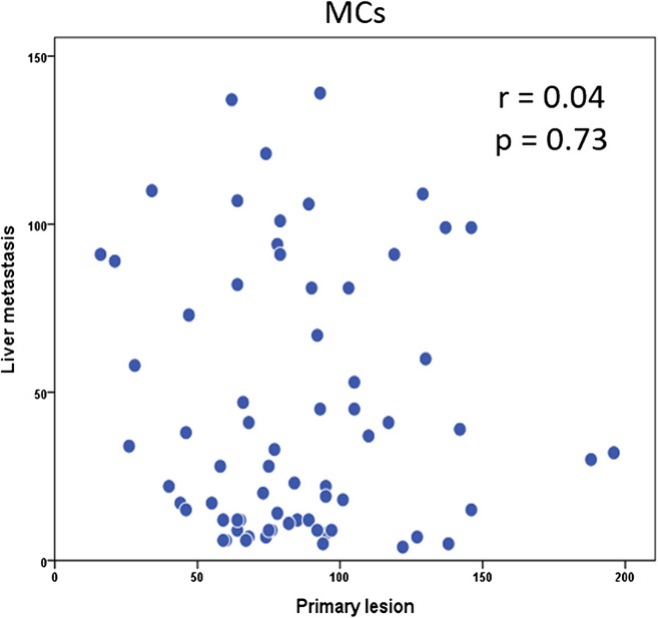
Tissue samples from resected primary lesions were stained with monoclonal antibodies to tryptase. The log-rank test was used to compare the number of mast cells (MCs) in primary lesions and metastatic lesions. There was no correlation between these parameters (*r* = 0.04, *p* = 0.73)

The distribution of cell numbers for each marker is shown in Table 1. The average number of TIMs were 84.9 in primary lesion and 42.2 in CRLM. The cutoff points selected using the maximal Youden index were 26 for peritumoral MCs, 176 for peritumoral Mφs, 17 for peritumoral DCs in CRLM. We found that 54 % (73 of 135) of patients were in the high peritumoral MC group. There was no significant relationship between number of peritumoral MCs and clinicopathological features, with the exception that the number of MCs in normal liver, the number of peritumoral Mφs, and microvessel density were significantly higher in the high peritumoral group than the low peritumoral group (*p* = 0.02, *p* = 0.04, and *p* < 0.01, respectively) (Table 1). There was no significant relationship between peritumoral Mφ or DC cell counts and clinicopathological features including number of peritumoral vessels (data not shown) or between the number of TIMs in primary lesions and liver metastasis (*r* = 0.04, *p* = 0.73; Fig. 2). Interestingly, MC counts were not changed regardless of whether oxaliplatin or bevacizumab is used.

### Number of TIMs and survival or recurrence

**Fig. 3:**
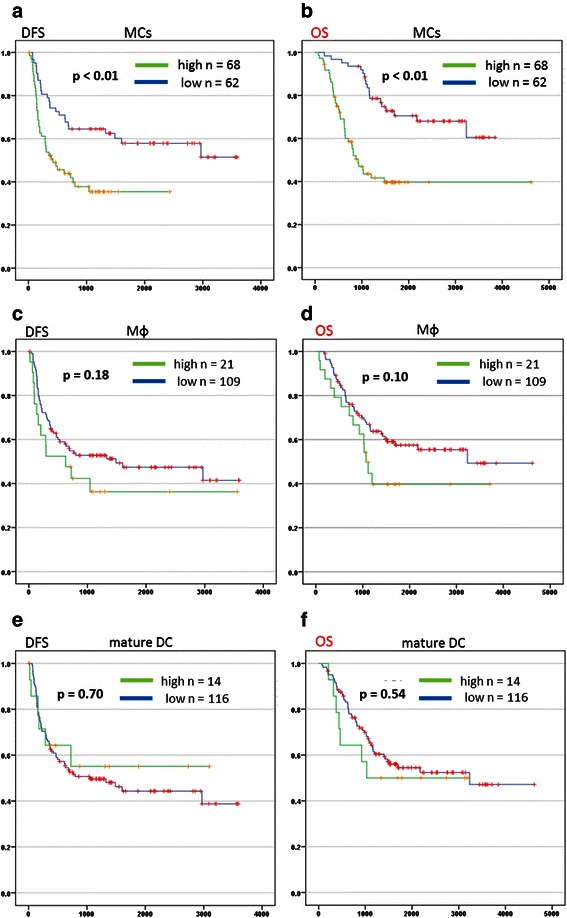
Tissue samples from resected metastatic lesions were stained with monoclonal antibodies to tryptase, MAC387 and CD83. (**a**, **c**, **e**) Disease-free survival (DFS) and (**b**, **d**, **f**) overall survival (OS) were determined using the Kaplan-Meier method, and the log-rank test was used to compare DFS and OS in groups with low and high numbers of peritumoral mast cells (MCs), peritumoral macrophages (Mφs) and peritumoral mature dendritic cells (DCs)

The DFS and OS curves for each peritumoral marker in CRLM are shown in Fig. 3. The 5-year DFS and OS rates were 21.6 and 38.1 %, respectively, in patients with high peritumoral MC numbers, and 42.6 and 55.6 %, respectively, in patients with low MC numbers (Fig. 3a, b). The differences in survival rates between the 2 groups were significant (*p* < 0.01 for both DFS and OS). On the other hand, there were no significant differences in DFS and OS between patients with low and high numbers of Mφs (*p* = 0.18 and 0.10, respectively) or mature DCs (*p* = 0.70 and 0.54, respectively) (Fig. 3c–f).

### Prognostic factors

Fifteen non-immunological and 3 immunological factors were included in the univariate analysis; these did not include treatment-related factors. Nine factors were significantly associated with OS including sex (male), synchronous hepatectomy, distribution of bilobar liver metastases, tumor number, extrahepatic disease, NAC, hypoalbuminemia, high level of pre-hepatectomy carcinoembryonic antigen, and a high peritumoral MC count. The factors with a p value of < 0.05 in the univariate analysis were further assessed in a multivariate analysis using a Cox proportional hazards model with stepwise selection. Hypoalbuminemia and high peritumoral MC infiltration were significant predictors of unfavorable OS in the multivariate analysis (Table 2).

**Table 2:** Univariate and multivariate analyses of overall survival

Variable	Univariate			Multivariate		
	*p* value	Hazard ratio	95 % CI	*p* value	Hazard ratio	95 % CI
Age, ≧70 years	0.39	0.7	0.3–1.6			
Sex, male	0.02	0.4	0.2–0.9	-	-	-
Timing of hepatectomy, synchronous	0.03	2.4	1.0–5.0	-	-	-
Distributution, biloblar	<0.01	3.4	1.5–7.5	-	-	-
Number of tumors, ≧4	<0.01	4.2	1.9–9.4	-	-	-
Diameter of tumors, ≧3	0.28	1.5	0.7–3.1			
Primary site, rectum	0.22	1.6	0.8–3.4			
Primary lymph node metastases, yes	0.91	1.0	0.5–2.3			
Extrahepatic disease, yes	<0.01	4.5	1.9–11	-	-	-
Neoadjuvant chemotherapy, yes	<0.01	3.9	1.7–8.5	-	-	-
CRP, ≧2 mg/L	0.2	2.3	0.6–7.9			
Alb, <3.5 g/dL	<0.01	6.4	1.6–25	<0.01	14.5	2.2–92
Lymphocytes, <1000	0.55	1.3	0.6–2.9			
Neutrophils, ≧6000	0.27	2.8	0.5–17			
CEA, ≧100 ng/mL	<0.01	3.3	1.4–8.0	-	-	-
Peritumoral MCs, high	<0.01	8.6	3.6–20	<0.01	17.3	4.8–62
Peritumoral Mφs, high	0.13	2.0	0.8–5.0			
Peritumoral mature DCs, high	0.34	1.7	0.6–5.3			

*CI* confidence interval, *CRP* C-reactive protein, *Alb* albumin, *CEA* carcinoembryonic antigen, *MCs* mast cells, *M*φ*s* macrophages, *DCs* dendritic cells

## Discussion

We demonstrated an association between the degree of peritumoral MC infiltration and clinical outcome following resection of CRLM. As shown via multivariate analysis, high peritumoral MC infiltration in CRLM was a significant independent predictor of unfavorable OS. On the other hand, high peritumoral MC infiltration in primary lesion was not significant independent predictor of unfavorable OS. We suggest that peritumoral MCs induce neo-angiogenesis and tumor cell proliferation. The prognostic significance of TIMs in colorectal cancer has been debated and is controversial [12, 13, 29, 43, 44].

Immunochemical results reflect the type of histological stain used and the area of the specimen analyzed. Because Giemsa and toluidine blue [29] are non-specific, we used anti-tryptase antibody to immunohistochemically detect tryptase-positive MCs. Tryptase activation promotes tumor cell proliferation and neo-angiogenesis by triggering the release of interleukin 6 and granulocyte-macrophage colony-stimulating factor from MCs. We thought that tumor-tumor microenvironment interaction could be evaluated by examining the peritumoral area rather than the tumor and therefore focused on this area in this study.

NAC sometimes affects the tumor microenvironment, for example, by causing lymphocyte infiltration as reported by Nakagawa et al. [10]. In our study, there was a significant difference in DFS and OS between patients with low and high MC counts regardless of whether they received NAC (data not shown). Therefore, determining infiltrating MC numbers is useful after resection of CRLM even in patients who did not receive NAC.

Consistent with previous studies [24, 29, 32], we found that high numbers of peritumoral MCs were associated with high numbers of microvessels in CRLM. The c-Kit pathway leads to MC activation and consequent expression of angiogenic cytokines (e.g., VEGF, PDGF, and FGF-2) and degranulation of MCs [19]. Roles of tumor-infiltrating Mφs and DCs in some cancers have been reported [39, 40, 45–48]. In this study, peritumoral Mφ and DC counts were not significant independent predictors of OS in patients with CRLM. Our data, however, must be interpreted in the context of its study design limitations. We recognize that tryptase is not the only MC marker in humans. Therefore, other MC markers such as chymase, histamine, heparin, VEGF, and PDGF-β should be tested via immunohistochemistry, and the relationship between the number of MCs identified via these markers and OS should be examined.

Ying et al. [49] reported that tumor cells promoted MC migration in pancreatic ductal adenocarcinoma, and SCF is a previously reported attractant of MCs [18]. Therefore, we need to clarify whether tumor cells produce SCF at metastatic lesions.

The relationship between TIM counts, angiogenesis, and tumor progression suggests that MCs may be a novel therapeutic target in CRLM. c-Kit tyrosine kinase inhibitors (e.g., imatinib and masitinib) may be used to block MC activation and degranulation, and tryptase inhibitors (e.g., gabexate and nafamostat mesylate) may be used to prevent the release of tryptase from MC cells [18, 50–52]. In the future, such drugs may improve the prognosis of patients with CRLM, particularly those with high MC infiltration. On the other hand, we did not find any relationship between the number of MCs in primary lesions and CRLM. The results of present study show that the number of MCs in metastatic lesions but not primary lesions is important for predicting the prognosis of CRLM patients and as an indication of therapy.

## Conclusions

The present study shows that the number of infiltrating peritumoral MCs is a significant predictor of unfavorable OS in patients who underwent hepatectomy for CRLM. Further study is required to identify the molecules that attract MCs to peritumoral sites in CRLM.

## Abbreviations

CRC: Colorectal cancer
CRLM: Colorectal liver metastases
DC: Dendritic cell
DFS: Disease-free survival
FGF-2: Fibroblast growth factor 2
Mφ: Macrophage
MC: Mast cell
NAC: Neoadjuvant chemotherapy
OS: Overall survival
PDGF: Platelet-derived growth factor
ROC: Receiver operating characteristic
SCF: Stem cell factor
TIM: Tumor-infiltrating mast cell
Treg: Peritumoral regulatory T cells
VEGF: Vascular endothelial growth factor
